# Supplementary material for: The S. Typhi effector StoD is an E3/E4 ubiquitin ligase which binds K48- and K63-linked diubiquitin
Source: Life Sci Alliance. 2019 May 29;2(3):e201800272. doi: 10.26508/lsa.201800272 (PMC6545606; doi:10.26508/lsa.201800272)
Supplement: Supplementary file 1 [file LSA-2018-00272_TableS1.docx]

Table S1. Strains used in this study.

| ***S.* Typhimurium Description Source or Reference** | | |
| --- | --- | --- |
| 14028  ICC796 | Wild-type *S.* Typhimurium  *S.* Typhimurium 14028 Δ*prgH* | ATCC  [1] |
| Δ*prgH*/Δ*ssaV* | *S.* Typhimurium 14028 Δ*prgH*::Tet and Δ*ssaV*::Kn (Tet^R^, Kn^R^) | [2] |

| ***S.* Typhi** | | |
| --- | --- | --- |
| Ty2 | Wild-type *S.* Typhi | Gordon Dougan |
| Δ*invA* | Ty2 Δ*invA*::Kn (Kn^R^) | [3] |
| Δ*ssaV* | Ty2 Δ*ssaV*::Kn (Kn^R^) | This study |
| Δ*stoD* | Ty2 Δ*stoD*::Kn (Kn^R^) | This study |

| ***Citrobacter rodentium*** | | |
| --- | --- | --- |
| ICC169 | Wild-type | [4] |

**References**

1. Johnson R, Byrne A, Berger CN, Klemm E, Crepin VF, Dougan G, et al. The type III secretion system effector SptP of *Salmonella enterica s*erovar Typhi. J Bacteriol. 2017;199: e00647-16. doi:10.1128/JB.00647-16

2. Beuzon CR, Unsworth KE, Holden DW, Beuzo CR, Holden DW. In vivo genetic analysis indicates that PhoP-PhoQ and the *Salmonella* pathogenicity island 2 type III secretion system contribute independently to *Salmonella* *enterica* serovar Typhimurium virulence. Infect Immun. 2001;69: 7254–7261.

3. Johnson R, Ravenhall M, Pickard D, Dougan G, Byrne A, Frankel G. Comparison of *Salmonella enterica* serovars Typhi and Typhimurium reveals typhoidal-specific responses to bile. Infect Immun. 2018;86: e00490-17. doi:10.1128/IAI.00490-17

4. Wiles S, Clare S, Harker J, Huett A, Young D, Dougan G, et al. Organ specificity, colonization and clearance dynamics in vivo following oral challenges with the murine pathogen *Citrobacter rodentium*. Cell Microbiol. 2004;6: 963–972. doi:10.1111/j.1462-5822.2004.00414.x
