# Supplementary material for: The S. Typhi effector StoD is an E3/E4 ubiquitin ligase which binds K48- and K63-linked diubiquitin
Source: Life Sci Alliance. 2019 May 29;2(3):e201800272. doi: 10.26508/lsa.201800272 (PMC6545606; doi:10.26508/lsa.201800272)
Supplement: Supplementary file 2 [file LSA-2018-00272_TableS2.docx]

Table S2. Plasmids used in this study.

| **Name** | **Description** | **Source or Reference** |
| --- | --- | --- |
| **Bacterial expression vectors** | | |
| pKD46 | Encoding lambda red recombinase (Amp^R^) | [1] |
| pKD4 | PCR template plasmid (Kn^R^) | [1] |
| pWSK29 | Low copy-number bacterial expression vector (Amp^R^) | [2] |
| pWSK29-Spec | pWSK29 with a spectinomycin resistance cassette | [3] |
| pWSK29-Spec-TEM1 | Encoding TEM1 (Spec^R^, Amp^R^) | This study |
| pWSK29-Spec-p*stoD*-4HA | Encoding *stoD* from the endogenous promoter (upstream 498 bp) with a C-terminal 4HA tag | This study |
| pET28a-*stoD* | Encoding *stoD* (Kn^R^) | This study |
| pET28a-*stoD*_P204K_ | Encoding *stoD*P_204K_ | This study |
| pET28a-*stoD* [1-95] | Encoding *stoD* amino acids 1-95 | This study |
| pET28a-*stoD* [1-101] | Encoding *stoD* amino acids 1-101 | This study |
| pET28a-*stoD* [134-233] | Encoding *stoD* amino acids 134-233 | This study |
| pET28a-*stoD*_P204K_ [134-233]  pET28a-*stoD_L167_*_A_ [134-233] | Encoding *stoD* amino acids 134-233 with a P204K mutation  Encoding *stoD* amino acids 134-233 with a L167A mutation | This study  This study |
| pET28b-*UBE2E1* | Encoding human *UBE2E1* with a N-terminal 6His tag (Kn^R^) | This study |
| pET3a-*ubiquitin* | Encoding full-length *ubiquitin* (Amp^R^) | [4] |
| pET3a-*ubiquitin_K48R_* | Encoding full-length *ubiquitin* with a K48R mutation | This study |
| pET3a-*ubiquitin_K63R_* | Encoding full-length *ubiquitin* with a K63R mutation | This study |
| pET3a-*ubiquitin_G76C_* | Encoding *ubiquitin* with a G76C mutation (Amp^R^) | This study |
| pJY2 | Helper plasmid encoding tRNA(AGA)(Arg) and T7 lysozyme (Cm^R^) | [5] |
| **Mammalian expression vectors** |  |  |
| pRK5 | Mammalian expression vector containing the SP6 promoter (Amp^R^) |  |
| pRK5-*mCherry* | pRK5 containing *mCherry* with an N-terminal HA tag | [6] |
| pRK5-*stoD*  pGFP-Rab11a  pGFP-Vamp3  pEGFP-Vamp3  pEGFP-LC3 | pRK5 containing *stoD* with a C-terminal HA tag  Encoding Rab11a with a GFP tag (Kn^R^)  Encoding Vamp3 with a GFP tag  Encoding LC3 with an EGFP tag | This study  [7]  [7]  [8] |

| ***S. cerevisiae* expression vectors** | | |
| --- | --- | --- |
| pGBKT7 | Yeast expression vector containing the Gal4 DNA-binding domain and the *ADH1* promoter (Amp^R^) | Clontech |
| pGBKT7-*stoD* | Encoding *stoD* fused to the Gal4 DNA-binding domain | This study |
| pGBKT7-*stoD* [1-133] | Encoding *stoD* amino acids 1-133 | This study |
| pGBKT7-*stoD* [134-233] | Encoding *stoD* amino acids 134-233 | This study |
| pGADT7 | Encoding the Gal4 activation domain and the *ADH1* promoter (Amp^R^). | Clontech |
| pGADT7-*Ubiquitin*  pGBKT7-NleG7  pGBKT7-NleG7_P177K_  pGBKT7-NleG-N  pGBKT7-NleG8  pGBKT8-NleG-N | Encoding a synthetic sequence of *Ubiquitin* from *S. cerevisiae*  Encoding NleG7 fused to the Gal4  DNA-binding domain  Encoding NleG7_P177K_ fused to the Gal4  DNA-binding domain  Encoding NleG7 N-terminus (amino acids 1-97) fused to the Gal4 DNA-binding domain  Encoding NleG8 fused to the Gal4 DNA-binding domain  Encoding NleG7 N-terminus (amino acids 1-109) fused to the Gal4 DNA-binding domain | This study  This study  This study  This study  This study  This study |

**References**

1. Datsenko KA, Wanner BL. One-step inactivation of chromosomal genes in *Escherichia coli* K-12 using PCR products. Proc Natl Acad Sci U S A. 2000;97: 6640–5. doi:10.1073/pnas.120163297

2. Wang RF, Kushner SR. Construction of versatile low-copy-number vectors for cloning, sequencing and gene expression in Escherichia coli. Gene. 1991;100: 195–9. Available: http://www.ncbi.nlm.nih.gov/pubmed/2055470

3. Johnson R, Byrne A, Berger CN, Klemm E, Crepin VF, Dougan G, et al. The type III secretion system effector SptP of *Salmonella enterica* serovar Typhi. J Bacteriol. 2017;199: e00647-16. doi:10.1128/JB.00647-16

4. Pickart CM, Raasi S. Controlled synthesis of polyubiquitin chains. Methods in Enzymology. Academic Press; 2005. pp. 21–36. doi:https://doi.org/10.1016/S0076-6879(05)99002-2

5. You J, Cohen RE, Pickart CM. Construct for high-level expression and low misincorporation of lysine for arginine during expression of pET-encoded eukaryotic proteins in Escherichia coli. Biotechniques. 1999/11/26. 1999;27: 950–954.

6. Harding CR, Stoneham CA, Schuelein R, Newton H, Oates C V, Hartland EL, et al. The Dot/Icm effector SdhA is necessary for virulence of *Legionella pneumophila* in Galleria mellonella and A/J mice. Infect Immun. American Society for Microbiology (ASM); 2013;81: 2598–605. doi:10.1128/IAI.00296-13

7. Clements A, Stoneham CA, Furniss RCD, Frankel G. Enterohaemorrhagic *Escherichia coli* inhibits recycling endosome function and trafficking of surface receptors. Cell Microbiol. Wiley-Blackwell; 2014;16: 1693. doi:10.1111/CMI.12319

8. Dupont N, Lacas-Gervais S, Bertout J, Paz I, Freche B, Van Nhieu GT, et al. *Shigella* phagocytic vacuolar membrane remnants participate in the cellular response to pathogen invasion and Are regulated by autophagy. Cell Host Microbe. Cell Press; 2009;6: 137–149. doi:10.1016/J.CHOM.2009.07.005
