# Supplementary material for: The S. Typhi effector StoD is an E3/E4 ubiquitin ligase which binds K48- and K63-linked diubiquitin
Source: Life Sci Alliance. 2019 May 29;2(3):e201800272. doi: 10.26508/lsa.201800272 (PMC6545606; doi:10.26508/lsa.201800272)
Supplement: Supplementary file 3 [file LSA-2018-00272_TableS3.docx]

Table S3. Primers used in this study - StoD.

| **Name** | **5’ to 3’ nucleotide sequence** |
| --- | --- |
| **pWSK29** | |
| BamHI StoD FW | CGCGGATCCAGAAGGAGATATACCTACGTAATGTTCTTAACATTTCCTAATGTAG |
| EcoRI StoD REV | CCGGAATTCTTATGCATAATCAGGCACATCATACGGATAAGCGTAGTCTGGGACGTCGTATGGGTATGGGATTATAAAATTACCTTTGG |
| pWSK29-Spec |  |
| pWSK29-Spec FW  pWSK29-Spec REV  TEM1 EV FW  TEM1 EV REV | GATATCAAGCTTATCGATACCG  AATTCCTGCAGCCCGGGGGA  TCCCCCGGGCTGCAGGAATTCGCACCCAGAAACGCTGGTG  GTATCGATAAGCTTGATATCTTACCAATGCTTAATCAGTGAGGCACC |
| SacI StoD with promoter FW | GGAGCTCCCAGATTTTCCCTGATGCC |
| PacI StoD with promoter REV | GCTTAATTAATGGGATTATAAAATTACCTTTGG |
|  |  |
|  |  |
| NotI Ty2 SopD FW  EcoRI Ty2 SopD REV | CATGGCGGCCGCATGCCAGTCACTTTAAGCTTCG  CGGAATTCCGTGTCAGTAATATATTACGACTG |
|  |  |
|  |  |
| BamHI StoD-TEM1 FW | GCCGGATCCAAGAAGGAGATATACCTACGTAATGTTCTTAACATTTCCTAATGTAGC |
| EcoRI StoD-TEM1 REV | CCGCCGGGAATTCCGTGGGATTATAAAATTACCTTTGG |
| **pET28a** | |
| BamHI StoD FW | CGCGCGGATCCATGTTCTTAACATTTCCTAATGTAGC |
| NotI StoD REV | GAATGCGGCCGCTTATGGGATTATAAAATTACCTTTGG |
| BamHI StoD [134-233] FW | CGCGCGGATCCATGAATAAACAACAATTAGTGGATAAGATTG |
| NotI StoD [134-233] REV | GAATGCGGCCGCTTATGGGATTATAAAATTACCTTTGG |
| Deletion T7 tag from pET28a *stoD* FW | GTGCCGCGCGGCAGCCATATGTTCTTAACATTTCCTAATGTAGCAATAACTCGAGATAAC |
| Deletion T7 tag from pET28a *stoD* REV | GTTATCTCGAGTTATTGCTACATTAGGAAATGTTAAGAACATATGGCTGCCGCGCGGCAC |
| Deletion residues 102-133 from *stoD* FW | CTGGACTGAATGTGTCGCGTGGATGAGCGGCCGCACTCGAGCACCAC |
| Deletion residues 102-133 from *stoD* REV | GTGGTGCTCGAGTGCGGCCGCTCATCCACGCGACACATTCAGTCCAG |
| Deletion residues 96-101 from *stoD* FW | CGCTATAAGGTTAGAACGACAATTAAATACTGGATGAGCGGCCGCACTCGAGCACCAC |
| Deletion residues 96-101 from *stoD* REV  BspEI StoD L167A Fw  BspEI StoD L167A Rev  KasI StoD P204K Fw  KasI StoD P204K Rev | GTGGTGCTCGAGTGCGGCCGCTCATCCAGTATTTAATTGTCGTTCTAACCTTATAGCG  GAAAAAGGCGTTTTTGTAAAAACTGGGCCAG  CGGAACAACTGCCATTACCGGGCATGTTAAGT  CACACATGCAGTTAGTAGGGCTCCATTATCAC  GCGCCGTCAATAATAAGTTGGATAAGTG |
| **pET3a-Ubiquitin** | |
| Ubiquitin G76C FWD | CTTGTCTTAAGACTAAGAGGTTGTTGAGGATCCGGCTGCTAAC |
| Ubiquitin G76C REV | GTTAGCAGCCGGATCCTCAACAACCTCTTAGTCTTAAGACAAG |
| Ubiquitin K48R FWD | CAAAGATTGATCTTTGCCGGTCGCCAGCTCGAGGACGGTAGAACGC |
| Ubiquitin K48R REV | GCGTTCTACCGTCCTCGAGCTGGCGACCGGCAAAGATCAATCTTTG |
| Ubiquitin K63R FWD | CGCTGTCTGATTACAACATTCAGCGCGAGTCGACCTTACATCTTGTC |
| Ubiquitin K63R REV | GACAAGATGTAAGGTCGACTCGCGCTGAATGTTGTAATCAGACAGCG |
| **pRK5** | |
| BamHI StoD FW | CGCGCGGATCCCGGCCACCATGGCGATGTTCTTAACATTTCCTAATGTAGC |
| HindIII StoD REV | GCCAAGCTTTTAAGCGTAGTCTGGGACGTCGTATGGGTAACCTGCTCCGAATTCTGGGATTATAAAATTACCTTTGGTAG |
| **pGBKT7** | |
| EcoRI StoD FW | CCGCCGGGAATTCATGTTCTTAACATTTCCTAATGTAGC |
| PstI StoD REV | TGCACTGCAGATTATGGGATTATAAAATTACCTTTGG |
| EcoRI StoD [1-133] FW | CCGCCGGGAATTCATGTTCTTAACATTTCCTAATGTAGC |
| PstI StoD [1-133] REV | TGCACTGCAGATCAAGATGTATCAAAAATAGCAGGTGCTCC |
| EcoRI StoD [134-233] FW | GCCGAATTCAATAAACAACAATTAGTGGATAAG |
| BamHI StoD [134-233] REV | GCCGGATCCTTATGGGATTATAAAATTACCTTTGG |
| EcoRI Ubiquitin FW | GCCGAATTCATGCAGATCTTCGTCAAGACG |
| BamHI Ubiquitin REV | GCCGGATCCTCAACCACCTCTTAGTCTTAAG |
| EcoRI UBE2L3 FW | GCCGAATTCATGGCGGCCAGCAGGAGG |
| BamHI UBE2L3 REV | GCCGGATCCTTAGTCCACAGGTCGCTTTTC |

| **Generating mutants in *S.* Typhi Ty2** | |
| --- | --- |
| SsaV KanR FW | GGCGCAACAGTGGCTCAGTGTATGCGCGGGTCGTCAGGATATGGTTCTGGTGTGTAGGCTGGAGCTGCTTCG |
| SsaV KanR REV | GCTAAGGTCAATACTTTCTACCACTTGTATAAGGCTCTCCTCTCCTAATTCATATGAATATCCTCCTTAG |
| SsaV Confirmation FW | GCGCGATGGGCTTGCAATG |
| SsaV Confirmation REV | CACGCATTTAACTTCGTTGCGC |
| StoD KanR FW | CCTAATGTAGCAATAACTCGAGATAACAGGATAGACAAATTATCTGAAAATGTGTGTAGGCTGGAGCTGCTTCG |
| StoD KanR REV | ATTACCTTTGGTAGTGTCAAAATAACATTCATTTTTATTTATTATCATATCCATATGAATATCCTCCTTAG |
| StoD Confirmation FW | GTCTTAATCTGTACAAAGGC |
| StoD Confirmation REV | CAACATCGAAACATAGACTGC |
