# Supplementary material for: The S. Typhi effector StoD is an E3/E4 ubiquitin ligase which binds K48- and K63-linked diubiquitin
Source: Life Sci Alliance. 2019 May 29;2(3):e201800272. doi: 10.26508/lsa.201800272 (PMC6545606; doi:10.26508/lsa.201800272)
Supplement: Supplementary file 4 [file LSA-2018-00272_TableS4.docx]

Table S4. Primers used in this study – NleG

**_________________________________________________________________________**

**Name 5’ to 3’ nucleotide sequence**

**­­­­­­­­­­­­_________________________________________________________________________**

EcoR1 NleG7 FW CGCCGGAATTCATGTCTTTAAATATAGGAT

GGCAAAAAG

EcoR1 NleG7 REV CGCCGGAATTCTCAGTCTGTATCCAGTATCCTGAAAT

GCTGATTCATAATATC

*silent mutation introduced to eliminate EcorI site within C-term on NleG7

EcoR1 NleG7-N-term-R CGCCGGAATTC**TCA**CAATTGAAGCATAAA

ATCTCGAG

EcoR1 NleG8 FW CGCCGGAATTCATGCCTATTATGTTAAATTTCTCAA

ATG

EcoR1 NleG8 REV CGCCGGAATTCTCAAAGACGCGTATCCGAC

EcoR1 NleG8-N-term-R CGCCGGAATTATTTCTGACACTTTCCTGCAAAG

NleG7 P177K FW CTTAGCAGGGAAAAGATAGCAA

NleG7 P177K REV CTTATGGGGGTGGTGCTTTCTG
