# Supplementary material for: The S. Typhi effector StoD is an E3/E4 ubiquitin ligase which binds K48- and K63-linked diubiquitin
Source: Life Sci Alliance. 2019 May 29;2(3):e201800272. doi: 10.26508/lsa.201800272 (PMC6545606; doi:10.26508/lsa.201800272)
Supplement: Supplementary file 5 [file LSA-2018-00272_TableS5.docx]

Table S5. Antibodies used in this study.

| **Antibody** | | **Origin** | **Dilution** | | **Source** | |
| --- | --- | --- | --- | --- | --- | --- |
|  |  |  | **Immunofluorescence** | **Western blot** |  |  |
| **Primary antibodies** | | | | | | |
| DnaK clone 8E2/2 | | Mouse | - | 1:10,000 | Enzo Life Sciences | |
| HA | | Rabbit | 1:500 | 1:1,000 | Sigma | |
| HA.11 clone 16B12 | | Mouse | 1:1,000 | - | Cambridge Bioscience | |
| Myc | | Mouse | 1:200 |  | Millipore | |
| Ubiquitin clone FK2 | | Mouse | 1:200 | 1:1,000 | Enzo Life Sciences | |
| NEDD8 | | Rabbit | 1:200 | - | Cell Signalling Technology | |
| SUMO-1 | | Rabbit | 1:100 | - | Cell Signalling Technology | |
| SUMO-2/3 | | Rabbit | 1:100 | - | Cell Signalling Technology | |
| GFP | | Rabbit | 1:1,000 | - | Abcam | |
| **Antibody** | **Origin** | | **Dilution** | | | **Source** |
|  |  |  | **Immunofluoresence** | **Western blot** | |  |
| **Secondary antibodies** | | | | | | |
| Anti-rabbit HRP | | Goat | - | 1:50,000 | Jackson Immunoresearch | |
| Anti-mouse HRP | | Goat | - | 1:1,000 | Jackson Immunoresearch | |
| Anti-mouse Alex Fluor-488 | | Donkey | 1:200 | - | Jackson Immunoresearch | |
| Anti-mouse RRX | | Donkey | 1:200 | - | Jackson Immunoresearch | |
| Anti-rabbit Alexa Fluor-488 | | Donkey | 1:200 | - | Jackson Immunoresearch | |
| Anti-rabbit RRX | | Donkey | 1:200 | - | Jackson Immunoresearch | |
